# CoLiDe: Combinatorial Library Design tool for probing protein sequence space

**DOI:** 10.1093/bioinformatics/btaa804

**Published:** 2020-09-21

**Authors:** Vyacheslav Tretyachenko, Václav Voráček, Radko Souček, Kosuke Fujishima, Klára Hlouchová

**Affiliations:** Department of Cell Biology, Faculty of Science, Charles University, Biocev, Prague, Czech Republic; Department of Biochemistry, Faculty of Science, Charles University, 128 00 Prague 2, Czech Republic; Department of Cybernetics, Center for Machine Perception, Faculty of Electrical Engineering, Czech Technical University in Prague, 166 27 Prague, Czech Republic; Institute of Organic Chemistry and Biochemistry IOCB Research Centre & Gilead Sciences, Academy of Sciences of the Czech Republic, 166 10 Prague, Czech Republic; Earth-Life Science Institute, Tokyo Institute of Technology, Tokyo 1528550, Japan; Department of Cell Biology, Faculty of Science, Charles University, Biocev, Prague, Czech Republic; Institute of Organic Chemistry and Biochemistry IOCB Research Centre & Gilead Sciences, Academy of Sciences of the Czech Republic, 166 10 Prague, Czech Republic

## Abstract

**Motivation:**

Current techniques of protein engineering focus mostly on re-designing small targeted regions or defined structural scaffolds rather than constructing combinatorial libraries of versatile compositions and lengths. This is a missed opportunity because combinatorial libraries are emerging as a vital source of novel functional proteins and are of interest in diverse research areas.

**Results:**

Here, we present a computational tool for Combinatorial Library Design (CoLiDe) offering precise control over protein sequence composition, length and diversity. The algorithm uses evolutionary approach to provide solutions to combinatorial libraries of degenerate DNA templates. We demonstrate its performance and precision using four different input alphabet distribution on different sequence lengths. In addition, a model design and experimental pipeline for protein library expression and purification is presented, providing a proof-of-concept that our protocol can be used to prepare purified protein library samples of up to 10^11^–10^12^ unique sequences. CoLiDe presents a composition-centric approach to protein design towards different functional phenomena.

**Availabilityand implementation:**

CoLiDe is implemented in Python and freely available at https://github.com/voracva1/CoLiDe.

**Supplementary information:**

Supplementary data are available at *Bioinformatics* online.

## 1 Introduction

Considering the vastness of the potential protein sequence space, naturally occurring proteins are constructed from a small number of coding sequences that arrange into a limited number of structural folds. While there are 20^100^ possible combinations for the design of a 100-amino-acid protein within the canonical amino acid alphabet, only ∼10^15^ sequences encode all proteins on Earth (Luisi, 2006). Furthermore, these sequences are estimated to fold into only ∼2000 distinct topologies (Govindarajan *et al.*, 1999). These observations raise numerous questions in the fields of biotechnology, synthetic biology and evolutionary biology: How easily can a useful sequence be encountered in the unexplored sequence space? Are there protein folds and functions outside those formed by the natural sequence pool?

Several recent studies have started providing answers to these questions. Both secondary and tertiary structures seem to be abundant in completely random sequences (Chiarabelli *et al.*, 2006; Davidson and Sauer, 1994; LaBean *et al.*, 2011; Tretyachenko *et al.*, 2017). Novel folds and functions have been encountered in random and semi-random sequence libraries, and some researchers argue that protein function may be discovered by entirely stochastic means (Chao *et al.*, 2013; Donnelly *et al.*, 2018; Fisher *et al.*, 2011; Keefe and Szostak, 2001; Ravarani *et al.*, 2018). In addition, the bioactivity of and cellular response to random sequences has been actively discussed in association with de novo gene birth (Bornberg-Bauer and Heames, 2019; Neme *et al.*, 2017). While it seems that protein structure and function can be encountered in random sequence space, different biological functions have been associated with specific amino acid composition and hence physicochemical properties. For example, positively charged and aromatic amino acids are known to promote protein–RNA interaction, evolutionary early amino acids promote solubility and trends in amino acid composition have been related to phenomena such as protein disorder and liquid–liquid phase separation (Blanco *et al.*, 2018; Doi *et al.*, 2005; Newton *et al.*, 2019; Wang *et al.*, 2018; Vymětal *et al.*, 2019). Local residue composition is apparently what makes natural sequences stand out from randomness (Weidmann *et al.*, 2019). Overall, these studies highlight the importance of developing tools to probe the protein sequence space in a rational way.

Several approaches to constructing synthetic protein sequence libraries have been developed. The simplest is direct chemical synthesis of a peptide from amino acid precursors but has major restrictions in sequence length and conformational biases [reviewed in Jaradat (2018)]. Another approach is based on construction of a degenerate DNA template with subsequent expression. The template can be designed either using triplet codon as the minimal unit, where pre-synthesized triplets are linked together, or at the single nucleotide level. Although the former method can provide a library with unbiased amino acid distribution at each template position, the cost of the trinucleotide phosphoramidite precursors limits its widespread adoption in laboratory practice (Virnekas *et al.*, 1994). On the other hand, template synthesis at the nucleotide level is economically feasible and is offered by multiple commercial oligonucleotide synthesis companies. Using this approach, random libraries have been constructed from simple repeat of frequently used degenerate codons, such as NNN and NNK. The major drawback of NNN/NNK method for protein engineering is its high level of degeneracy (NNK codes 20 amino acids via 32 different codons). An elegant solution to reduce the degeneracy introduced by Kille *et al.* combines three degenerate codons in a vertical way to cover all 20 amino acids using 22 codons (so-called ‘22c-trick’) without an introduction of STOP codons (Kille *et al.*, 2013). Nevertheless, this solution is effective only when screening a few positions because of an increased cost of oligonucleotide synthesis (mere three mutagenized positions would demand 3^3^=27 separate oligonucleotides) and the experimental effort during template assembly. Both of these methods are focused on producing the highest mutational coverage without any attention to amino acid distribution of the mutant library.

While several computational algorithms for library design exist, they have been optimized to introduce as few degenerate codons as possible (Jacobs *et al.*, 2015; Shimko *et al.*, 2020; Tang *et al.*, 2012). An optimal solution to amino acid distribution approximation by combinations of degenerate codons was recently introduced in SwiftLib and DeCoDe algorithms (Jacobs *et al.*, 2015; Shimko *et al.*, 2020). Both produce compact combinatorial libraries by as few degenerate codons as possible while DeCoDe implements complex patterns of covariation into the library design (Shimko *et al.*, 2020). Degenerate codon positions consist of nucleotide mixtures at equimolar ratios where more than one nucleotide is found at a single position. An alternative approach is represented by use of spiked codons where nucleotides can be represented by variable ratios. Mapping of amino acid distribution into a single spiked codon was implemented by Wolf *et al.* and Craig *et al.* via numerical optimization and genetic algorithms. Unfortunately neither of these algorithms is publicly available (Craig *et al.*, 2009; Wolf and Kim, 2008). Although these tools are particularly useful for site-specific randomization strategies, there remains a missed opportunity for the overall design of protein libraries. Specifically, the formation of combinatorial segments of versatile length with a desired amino acid composition would benefit synthetic biology practitioners.

Here, we present a combinatorial library design tool (CoLiDe) for the DNA template design of versatile protein libraries. CoLiDe aids in construction of libraries with specific amino acid distributions and lengths, i.e. optimization of the overall amino acid composition. Such libraries are notably in demand for investigating phenomena that are principally related to amino acid composition—protein liquid–liquid phase separation (Wang *et al.*, 2018), intrinsic protein disorder (Vymětal *et al.*, 2019), spatial protein localization in vivo (Cedano *et al.*, 1997), protein degradation half-life in the cellular milieu and chain elongation rate during ribosomal synthesis (Guruprasad *et al.*, 1990; Riba *et al.*, 2018). In addition, our algorithm allows for incorporation of spiked trinucleotides (i.e. with variable nucleotide composition for single position) and removal of specific codons, such as for codon reassignment and incorporation of unnatural amino acids (Liu and Schultz, 2010).

As a proof-of-concept, we demonstrate the use of CoLiDe by construction of a combinatorial protein library of 33 amino acids in length and composed of a 10 amino acid alphabet (A, S, D, G, L, E, T, I, P and V). Total amino acid composition of the library and therefore each protein sequence was specified using the CoLiDe input option. Moreover, CoLiDe can be used to upgrade currently available DNA block shuffling methods to prepare combinatorial libraries that are hundreds of amino acids in length.

## 2 Materials and methods

### 2.1 CoLiDe algorithm

#### 2.1.1 Basic definitions

The following procedure addresses problem-solving with spiked codons (degenerate codons with variable nucleotide composition). If the domain is restricted to degenerate codons, the procedure differs slightly, as noted below. We considered spiked codon to be a 12-tuple concatenated from 4-tuples representing each degenerated position of the triplet:

$$\left(T_{1},C_{1},A_{1},G_{1},T_{2},C_{2},A_{2},G_{2},T_{3},C_{3},A_{3},G_{3}\right)$$

satisfying

$$\begin{array}{c} \forall i\in \left\{1,2,3\right\}:T_{i}+C_{i}+A_{i}+G_{i}=1\\ \forall i\in \left\{1,2,3\right\}\forall S\in \left\{T,C,A,G\right\}:S_{i}\geq 0. \end{array}$$

We also introduced a 12-tuple base-codon term:

$$\left(T_{1},C_{1},A_{1},G_{1},T_{2},C_{2},A_{2},G_{2},T_{3},C_{3},A_{3},G_{3}\right)$$

satisfying

$$\begin{array}{c} \forall i\in \left\{1,2,3\right\}:T_{i}+C_{i}+A_{i}+G_{i}\geq 1\\ \forall i\in \left\{1,2,3\right\}\forall S\in \left\{T,C,A,G\right\}:S_{i}\in \{0,1\}. \end{array}$$

Base-codons serve as templates for codons. For example, the codon NNS can be represented by the 12-tuple (1,1,1,1,1,1,1,1,0,1,0,1), meaning that the first two positions can include all four bases and the last position is restricted to C or G only. By defining base-codon b, a spiked codon can be obtained by replacing 1’s in b with non-zero numbers. Note that in cases of restriction to degenerate codons, there is one-to-one mapping between degenerate codons and base-codons.

The optimization problem can be formulated as follows: given amino acid sequence length l; desired amino acid distribution D, which is a vector of 21 non-negative numbers summing up to 1, one number for each amino acid; a set of forbidden codons F; and a distance function dist, find a multiset M cardinality l of codons, minimizing dist(D,M), subject to $\forall m\in M\forall f\in F\exists p:f_{p}\neq 0\Rightarrow m_{p}=0$, where $f_{p}$ is an element of f on position p. This condition guarantees that there are no forbidden codons in M.

Every codon encodes a distribution of amino acids. Hence, M representing a multiset of degenerate codons, can be considered as a mixture distribution of amino acids encoded by its codons. The closer the mixture distribution encoded by M is to D, the smaller dist(D,M) should be. We defined D as a vector in $R^{21}$, so that we could use a norm to measure the distance between two distributions. Common norms include the $L^{1}$ norm, which is a sum of absolute values of elements, and the $L^{2}$ norm, which is a square root of the sum of squares of elements. As square root is a strictly increasing function, minimizing the square root of a sum of squares and minimizing a sum of squares yield the same optimal argument. The third common norm is the $L^{\infty }$ norm, which is the greatest absolute value of elements. We used the $L^{2}$ norm in our implementation, as it penalizes large differences considerably but is permissive for slight deviations.

**Algorithm**

We present the base implementation of the CoLiDe algorithm as a pseudocode:

1. *BC* ← generate valid base-codons
2. *M* ← ∅
3. For *i *=* *1 to *l*:
  - *bc* ← random element from *BC*
  - *c* ← make random codon from *bc*
  - *M* ← *M* ∪ {*c*}
4. *rejected* ← 0
5. While *rejected* < 1000 · l:
  - *bc* ← random element from *BC*
  - *c* ← make random codon from *bc*
  - d_old_ ← *dist(D, M)*
  - *M*2 ← *M* ∪ {*c*} \ (random element from *M*)
  - *d*_new_ ← *dist(D, M*2*)*
  - If *d*_new_ < *d*_old_
    - *M* ← *M*2
    - *rejected* ← 0 Else i. *rejected* ← *rejected* + 1
6. Output *M*

In the first step, valid base-codons are generated. There are 3 independent sequences in base-codon $\left(T_{i},C_{i},A_{i},G_{i},i\in \{1,2,3\}\right)$, and every sequence is an arbitrary binary string of length 4, excluding string 0000. There are 16 − 1 such strings, so the number of base codons is (16 − 1)^3^=3375. Along the fact that there are at most 64 forbidden codons, the time needed to execute this step is negligible with any reasonable implementation.

In the third step, filling multiset M with random codons yields an initial result.

In the fifth step, the optimization is performed. Once per loop, a random codon is generated, and an attempt is made to replace a random codon in M with this codon. If the objective improves, the change is accepted; otherwise, it is rejected. The algorithm works reasonably well and reasonably quickly (visualization of results is many times slower than the algorithm itself). The base algorithm can be easily modified, because *dist* can be chosen arbitrarily. In our implementation, *dist* is chosen as the $L^{2}$ norm of the vector of differences between D and the distribution of amino acids encoded by codons of M. This problem also could be formulated as a quadratic programming task, but it would be difficult or even impossible to add new requirements to the result. The ability of the algorithm to be easily extended to new problems offers flexibility.

### 2.2 Library construction

#### 2.2.1 Preparation of DNA and RNA templates

A degenerate ssDNA of 197 bases was synthesized by Integrated DNA Technologies (Supplementary Material Sequences, library). The oligonucleotide was converted to dsDNA by Klenow extension with a 5′ complementary reverse primer (Supplementary Material Sequences, reverse). Annealing of the primer was performed by cooling down a mixture of 2 μM oligonucleotide and primer in the presence of 200 μM dNTPs in buffer NEB1 from 90 to 25°C at a rate of 1°C/min. Total 10 U Klenow polymerase was added to the annealed mixture, and extension step was carried out for 1 h at 37°C followed by polymerase deactivation at 50°C for 15 min. The dsDNA library product was purified with the Monarch^®^ PCR & DNA Cleanup Kit (New England Biolabs) and used for the downstream in vitro transcription, carried out with the Ampliscribe T7-Flash kit (Lucigen) according to the manufacturer’s recommendations. The resulting mRNA was purified by ammonium acetate precipitation and dissolved in RNase free water to a final concentration of 3 µg/µl.

#### 2.2.2 cDNA preparation for high-throughput sequencing (HTS)

Complementary DNA (cDNA) was prepared from 1 µg transcribed mRNA. cDNA was synthesized according to the SuperScript IV (Thermo Fisher Scientific) instruction manual using reverse primer (Supplementary Material Sequences, reverse) and 20 μl reverse transcribed product was further amplified with Q5 DNA polymerase (New England Biolabs) in a 100-µl reaction volume for 11 amplification cycles with a primer annealing temperature of 68°C.

#### 2.2.3 Protein expression and purification for amino acid analysis and mass spectrometry

The protein library was prepared in a PUREfrex 2.0 (GeneFrontier Corporation) cell-free protein expression system. The reaction was prepared according to the manufacturer’s recommendations, supplemented with 0.05% Triton X-100 (v/v), and initiated by addition of 3 µg library mRNA. Protein expression was conducted for 4 h at 30°C. The reaction was diluted 10 times with guanidine denaturation buffer (6 M guanidine hydrochloride, 100 mM sodium phosphate, 500 mM NaCl, 0.05% Triton X-100, pH 8) and incubated with 4 µl TALON affinity chromatography resin (Clontech) for 12 h at 25°C. The resin was washed twice with urea denaturation buffer (8 M urea, 100 mM sodium phosphate, 500 mM NaCl, 0.05% Triton X-100, pH 8) and twice with distilled water supplemented with 0.05% Triton X-100. The library was eluted by boiling the affinity matrix in 50 µl of 2% (w/v) aqueous SDS. Eluted fractions were purified from SDS by addition of 5× volumes of ice-cold acetone. The precipitates were centrifuged, washed with 100% acetone and air-dried.

#### 2.2.4 Preparation of libraries for HTS and data analysis

The dsDNA library template was analyzed by HTS with an Illumina MiSeq. Prior to sequencing the library preparation, quantification was carried out on a Quantus™ Fluorometer (Promega). A total of 100 ng of DNA sample was used as an input for library preparation with the NEBNext Ultra II DNA Library Prep kit (New England Biolabs) with AMPure XP purification beads (Beckman Coulter). The length of the prepared library was determined with an Agilent 2100 Bioanalyzer (Agilent Technologies) and quantified with a Quantus Fluorometer (Promega). Samples were sequenced on a MiSeq Illumina platform using the Miseq Reagent Kit v2 for 500 cycles (2 × 250) in paired-end mode. Raw data was processed with Galaxy platform. Sequence analysis of assembled and filtered paired reads was performed with MatLab scripts developed by the Heinis lab (Afgan *et al.*, 2018; Rebollo *et al.*, 2014).

#### 2.2.5 Amino acid analysis and mass spectrometry

The purified and precipitated library samples were hydrolyzed in 6 M hydrochloric acid at 110°C for 20 h, the hydrolysate was evaporated, and reconstituted with 0.1 M hydrochloric acid containing the internal standard. Amino acid analysis was performed on an Agilent 1260 HPLC (Agilent Technologies) equipped with a fluorescence detector using automated o-phtalaldehyde/2-mercaptopropionic acid (OPA/MPA) derivatization. For mass spectrometry, the purified protein library sample was resuspended in water. The spectrum was collected after addition of 2,5-dihydroxybezoic acid matrix substance (Merck) using an UltrafleXtreme™ MALDI-TOF/TOF mass spectrometer (Bruker Daltonics, Germany) in linear mode.

## 3 Results and discussion

In this work, we present a computational tool for automated design of combinatorial libraries. CoLiDe uses evolutionary approach to find a satisfactory solution. The algorithm provides a set of degenerate codons which approximate the total amino acid distribution of protein without regard to individual degenerate positions in the coding template. The principle of the algorithm is summarized inFigure 1.

**Fig. 1.**
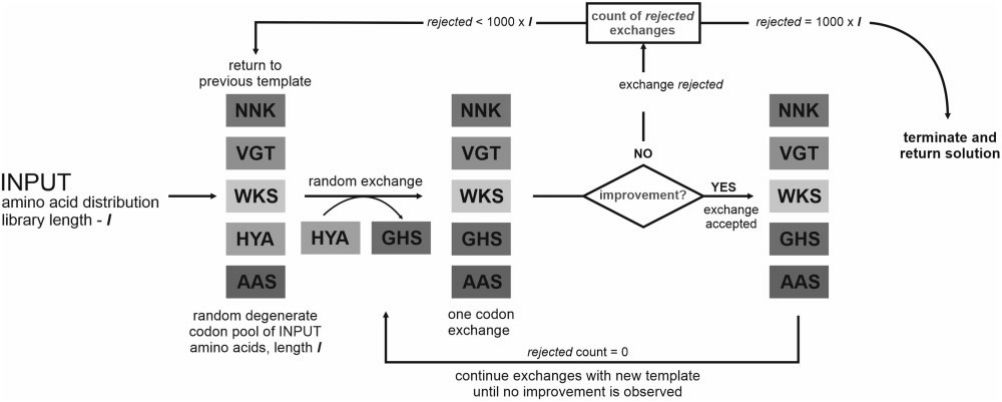
Outline of the CoLiDe algorithm. Based on the input amino acid distribution and length of the randomized library, at first an unoptimized vector of degenerate codons of given length is generated. Then the vector is optimized by single exchanges of codons until a vector of degenerate codons with minimal distance from the input distribution is obtained

Mandatory inputs include library length, amino acid distribution and degenerate codon type (standard or spiked, Supplementary Fig. S1). Other parameters, such as organism-specific codon preference, extent of degeneracy or codon removal/reassignment, also can be specified (Supplementary Fig. S1). Once the input parameters are defined, codons are pre-selected based on the amino acid input from a total pool of 3375 degenerate codons. The codon pre-selection removes undesired amino acid and STOP codons. This step guarantees that the combinatorial library is composed only of input amino acids and will not contain prematurely terminated templates. On the other hand, depending on input distribution, most highly degenerate codons are removed which reduces degeneracy of individual library positions.

Only the pre-selected degenerate codons serve in the subsequent library construction pipeline. The pipeline starts with random sets of degenerate codons of desired library length and follows with random codon exchanges (standard codons) or a shift in nucleotide ratios (spiked codons). Exchanges and shifts are kept within the optimized codon set if the amino acid product comes closer to input distribution (evaluated by mean squared error) and rejected if not. Optimization is finished when repeated changes do not further improve the solution (specifically, after *n* = 1000 × [library length] rejected mutations) This threshold was selected after test runs of the optimization path which recorded the rejection rate of mutations and provided satisfactory deviation on all tested distributions (Supplementary Fig. S2A–D). The output of the algorithm is a vector of degenerate codons of given library length. In other words, CoLiDe provides a list of degenerate codons combined randomly into a single oligonucleotide template.

CoLiDe offers a graphical user interface (Supplementary Fig. S1) that aids input of all variables, displays statistics of the optimized solution and allows the user to generate a report as a PDF document. CoLiDe is implemented in Python 3, and the source code is available as open source under MIT license at https://github.com/voracva1/CoLiDe.

### 3.1 CoLiDe performance analysis

**Fig. 2.**
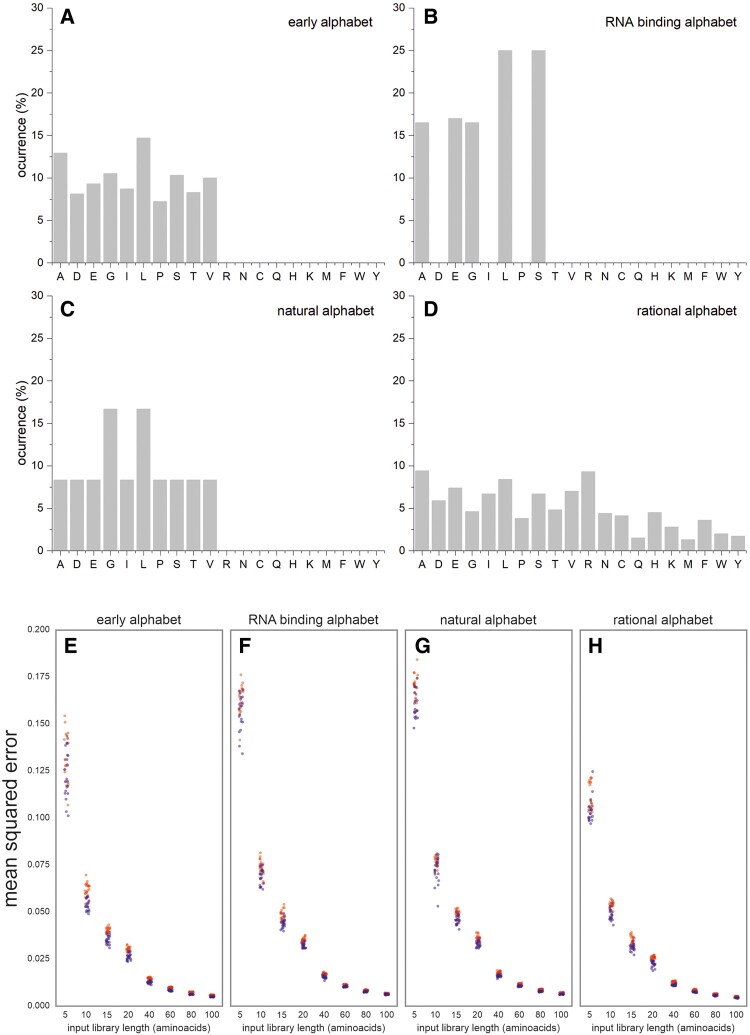
CoLiDe performance analysis. Amino acid distributions used to benchmark CoLiDe performance (**A–D**) and comparison of solutions generated from each (**E–H**). Each distribution was approximated *via* degenerate (red) and spiked (blue) codons. Solutions were produced in 10 replicates for various library lengths ranging from 5 to 100 amino acids

We tested CoLiDe’s precision and reproducibility on the following four amino acid distributions: (i) a reduced alphabet used in protein evolution studies to approximate an early version of the genetic code (Solis, 2019), (ii) a functional distribution derived from an analysis of RNA-binding proteins (Blanco *et al.*, 2018), (iii) a natural amino acid distribution from the UniProt database (UniProtKB/Swiss-Prot UniProt release 2019_11) and (iv) a rational selection of a reduced set of amino acids for protein engineering (Murphy *et al.*, 2000) (Fig. 2A–D and Supplementary Table S1). For each amino acid distribution, optimization was performed 10 independent times for library lengths of 5, 10, 15, 20, 40, 60, 80 and 100 amino acids (Fig. 2E–H). CoLiDe was able to reliably spread all the tested distributions on a DNA template of given length.

Mean squared errors in the shortest amino acid libraries ranged from 0.11 to 0.17 between individual alphabets and converged with increasing template length to values around 0.005. Variance in precision between solutions—measured as a coefficient of variation was highest in short libraries, ranging between 10^−2^ and 10^−3^, and decreased to values around 10^−5^ in longer templates (Supplementary Table S2).

Our results confirmed that the algorithm consistently finds precise solutions to selected input amino acid distributions. The precision of the solution increases and the variance between solutions within each group decreases along with the increase in library template length. With reduced template length, error became dependent on the specific amino acid alphabet. Solutions using spiked codons showed better precision with similar variance within each group (Supplementary Table S2). CoLiDe runtimes were tested on four library templates (Fig. 2A–D) with the template sizes ranging from 5 to 400 degenerate codons. Reported runtimes range from ∼3 to 600 s on Intel i5-8250U laptop (Supplementary Fig. S3).

Diverse degenerate libraries can be produced with other available tools, even though they are designed for construction of different library types. CoLiDe, in contrast to alternative design tools (SwiftLib, DeCoDe), focuses on combinatorial library design without position-specific restraints. Designed libraries are suitable for probing the constrained sequence space rather than for screening small, rationally designed library of protein variants (Jacobs *et al.*, 2015; Shimko *et al.*, 2020). As an example, we compare the solutions for combinatorial libraries provided by degenerate codon optimization algorithm SwiftLib (Jacobs *et al.*, 2015). SwiftLib outputs an optimized set of degenerate codons which cover the provided amino acid variability with as few degenerate codons as possible. Such approach faces difficulty to assure the precision of the distribution when targeting longer regions, whereas that is not the case for CoLiDe (Supplementary Fig. S5). On the other hand, SwiftLib outperforms CoLiDe when very short randomized regions (of 2–3 codons) are calculated (Supplementary Fig. S4). Deviations of ratios of single amino acids are reported in Supplementary Tables S4 and S5. CoLiDe provides a better choice for combinatorial design of longer protein templates provided that overall amino acid distribution of sequence is preferred over the specific amino acid variations on predefined positions. Furthermore CoLiDe can be used in protein engineering applications for coarse grained yet computationally efficient vertical design (multiple degenerate oligonucleotides per one tube) of degenerate codons to approximate amino acid distributions in single protein positions, similarly to established deterministic approaches described by Jacobs and coworkers (Jacobs *et al.*, 2015).

### 3.2 Proof-of-concept experimental library design

**Fig. 3.**
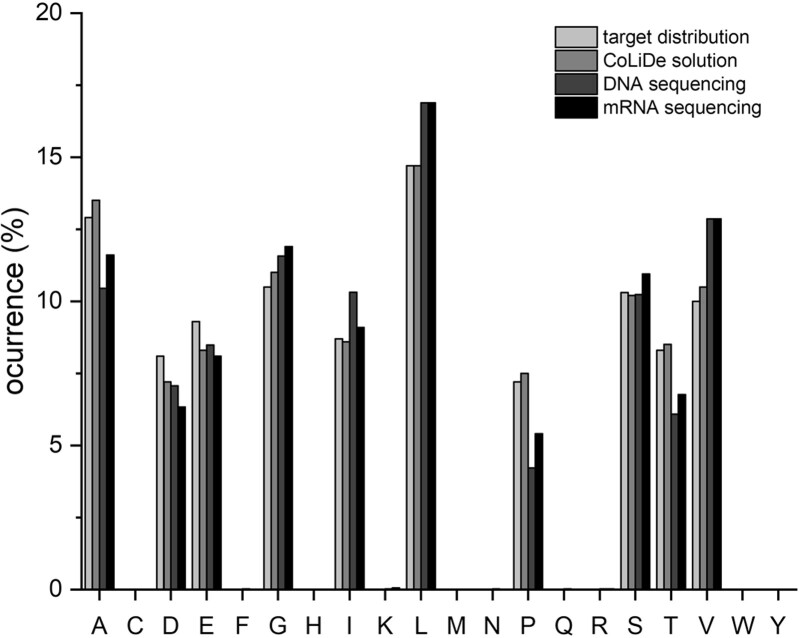
Comparison of the amino acid distribution of the CoLiDe solution of 33 amino acid long library to its target distribution and the DNA and mRNA templates obtained from the high-throughput sequencing (HTS) data (upon *in silico* translation)

To identify general pitfalls and experimental bottlenecks of library preparation, we experimentally evaluated one specific CoLiDe solution from DNA to protein level. A 45 amino acid protein library was prepared with a randomized region of 33 amino acids, following the early alphabet distribution (Fig. 2A). The mean squared error of the randomized region with CoLiDe solution was 0.0022 with an error variance of 0.00011 (Fig. 3). The random 33 codon region was tagged with an 8×H+QH (i.e. octa-His + Gln-His) coding sequence (separated by a two amino acid linker, KS) on the C-terminus for subsequent purification (Supplementary Material, Sequence). The protein coding sequence was embedded into a linear expression cassette, and the library was transcribed as described in Section 2 (Supplementary Fig. S6).

The length of the protein library was selected so that a single commercially synthesized oligonucleotide could be used for the downstream procedure. However, a larger construct could be prepared by DNA shuffling methods as previously described (Cho *et al.*, 2000). Thus, CoLiDe algorithm can also be utilized for the construction of random protein libraries with amino acids residues up to several hundreds.

### 3.3 Construction and characterization of the oligonucleotide library

**Fig. 4.**
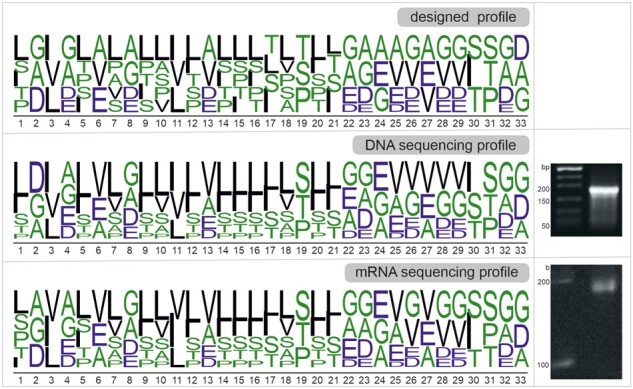
Preparation and analysis of DNA and RNA libraries. (left) Sequence logos generated *in silico* from the designed template (top), sequenced DNA template (middle) and sequenced reverse-transcribed mRNA (bottom). (right) Agarose gel electrophoresis of dsDNA library template (middle) and urea PAGE analysis of single stranded random library mRNA and (bottom). Polar and small amino acids (G, S, T, P, A) are green, hydrophobic and large amino acids are black (L, V, I) and negatively charged residues (D, E) are blue

Nucleotide sequences for degenerate libraries were analyzed on the DNA and mRNA template levels by high-throughput sequencing (HTS). The *in silico* translated amino acid composition (from both the DNA and mRNA templates) showed good agreement with the designed construct (Figs 3 and 4, Supplementary Table S6). While deviations of whole distributions are listed here as mean squared error calculated on (0,1) scale, we plot single amino acid occurrence as percentage of input distribution on (0,100) scale. Deviations between the CoLiDe solution and the in silico translated DNA template were observed in enrichment of valine, leucine and isoleucine (2.9, 2.2 and 1.6%) and depletion of proline, threonine and alanine (3, 2.2 and 2.4%) (Figs 3 and 4, Supplementary Table S6).

Upon analysis of nucleotide frequencies at each position, we found that deviation can be explained by the nucleotide composition bias during the oligonucleotide synthesis and have been confirmed as the current bottleneck by the provider (Supplementary Fig. S7). Statistical analysis of the sequencing data provides a confirmation of library diversity and shows that vast majority (99.9%) of all sequences are unique (Supplementary Table S7). Overall, mean squared error of amino acid distribution of DNA and RNA templates remained to be around ∼0.02 (Supplementary Table S6). Hence, we found that while CoLiDe algorithm can provide low mean squared error for the library design, one should be aware of the nucleotide bias that will be introduced during the oligonucleotide synthesis of highly degenerate DNA oligonucleotides. Such nucleotide composition bias of DNA library depends on each oligonucleotide provider (unpublished observation).

### 3.4 Construction and characterization of the protein library

**Fig. 5.**
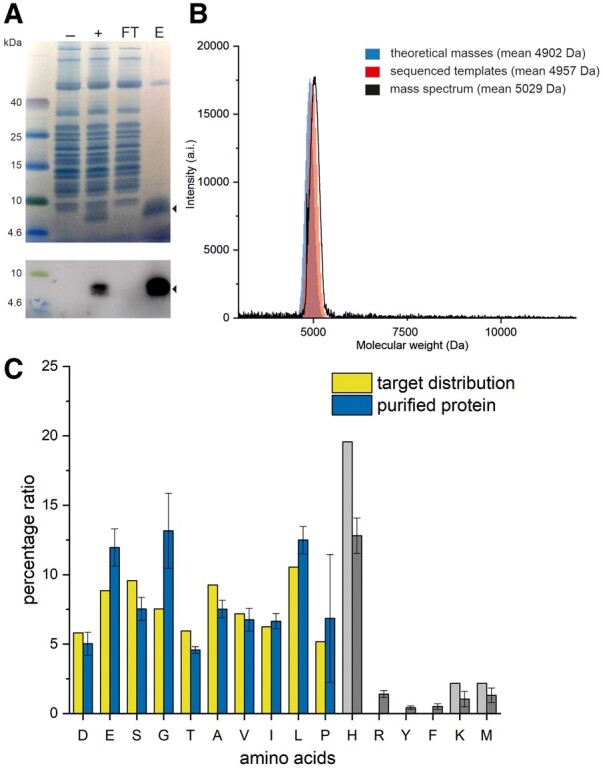
Preparation and analysis of the protein library. (**A**) SDS-PAGE and Western blot analysis of library expression and purification. The library was expressed in a recombinant cell-free system PUREfrex 2.0. ± stands for cell free fraction without and with expressed library, FT is affinity purification flow through and E is eluted fraction. (**B**) MALDI-TOF MS analysis of the purified library (black) compared with the theoretical mass distribution (blue) and mass distribution calculated from sequenced DNA templates (red). (**C**) Results of amino acid analysis deviations of variable (colored) and constant sequence regions/contaminations (grey) of the expressed and purified protein library in percentage units

The combinatorial protein library was expressed using an in vitro translation system and His-tag purified for downstream analysis (Fig. 5A). Expressed proteins were assessed by mass spectrometry (Fig. 5B) and amino acid analysis (Fig. 5C, Supplementary Table S6).

MALDI-TOF mass spectrometry revealed good agreement with expected values. The expected mass distribution was produced by analysis of 600 000 random sequences corresponding to the degenerate DNA template and by in silico translation of 600 000 sequences obtained by HTS of DNA and mRNA templates. The experimental spectrum is represented by normal weight distribution with a mean value of 5029 Da and a standard deviation of 120.6 (Fig. 5B). This is slightly shifted from the mean value of the molecular weight distribution expected from the design (4902 Da), partly as a result of sequence bias during the solid-state oligonucleotide synthesis. However, in silico translation of sequences obtained by HTS (producing a mean molecular weight of 4957 Da) confirms that this explains only part of the shift. This result indicates that the translation and purification steps have introduced additional compositional shift into the protein library. Most notably, the purified protein library is under-represented in alanine, aspartic acid and threonine (by 2–4% from the desired amount) and enriched in glutamic acid and glycine (by ∼5% from the input) as assessed by amino acid analysis (Fig. 5C), likely due to their impact on protein solubility and contamination by carry over protein components from the cell-free expression system in the purified library sample (Fig. 5A). While these deviations do not represent a major difference in the overall amino acid ratio profile [amino acid analysis shows an overall of 0.05 mean squared error (Supplementary Table S6)], it is important to be aware of the sequence biases that may be introduced into designed libraries during oligonucleotide synthesis and downstream procedures as a result of the translation and purification process or the physicochemical properties of the expressed proteins themselves.

Currently, there is no satisfactory methodology to analyze the variability of the large protein sequence pool directly. One translation reaction (in a 20 µl volume) is typically primed with 10^11^–10^12^ different template molecules. Even with the genotype-phenotype linked display methods (i.e. mRNA-display, ribosome display, etc.) number of characterized sequences is limited to the performance of HTS. Because neither DNA library preparation, RNA transcription nor the *in vitro* translation involve sequence amplification, a similar variability of protein sequences is expected after translation. The computational protocol therefore presents a tool for truly effective exploration of the protein sequence space.

## 4 Conclusions

Here, we present CoLiDe, a novel tool for precise design of combinatorial protein libraries of flexible length and desired amino acid composition. We provide evidence that it performs with minimal error and variance across several different amino acid distributions and lengths. It significantly outperforms SwiftLib (that have been developed for other applications) especially when designing combinatorial libraries longer than ∼10 amino acids.

In addition, we present a model protocol for combinatorial library (composed of a 10 amino acid alphabet) preparation by cell-free expression. By monitoring the DNA and mRNA sequence pool during library preparation using HTS, we confirmed the desired variability (99.9% of the sequences representing unique species). While negligible error is detected between the input sequence and the CoLiDe solution, up to 3% deviations of individual amino acid ratios were detected upon in silico translation of the mRNA sequence pool. The error was primarily attributable to nucleotide compositional bias from the synthesis of the starting material.

Using the template mRNA, we expressed and purified a highly variable protein library (represented by a normal weight distribution). To our knowledge, this is the first report of purification of a combinatorial protein library in an amount sufficient for biophysical characterization. The experimental procedure introduced additional detectable shifts among several amino acid compositions (up to 5% deviation), likely occurred during translation and purification steps of the library. Such an error is to be expected and may vary depending on the nature of individual amino acid alphabets. We estimate that 1011–1012 unique protein sequences can be produced in a 20-µl cell-free translation reaction using our protocol.

The design and experimental strategy presented here can be used in combination with vertical library design strategies (i.e. mixing multiple degenerate templates) and DNA shuffling synthesis. This represents a powerful tool for the synthesis of combinatorial protein libraries composed of hundreds of amino acids.

## Supplementary Material

btaa804_Supplementary_Data
