# Supplementary material for: CoLiDe: Combinatorial Library Design tool for probing protein sequence space
Source: Bioinformatics. 2020 Sep 21;37(4):482–9. doi: 10.1093/bioinformatics/btaa804 (PMC8088326; doi:10.1093/bioinformatics/btaa804)
Supplement: btaa804_Supplementary_Data [file btaa804_supplementary_data.zip › CoLiDe_suppl.docx]

CoLiDe: Combinatorial Library Design tool for probing protein sequence space

Vyacheslav Tretyachenko^1,4^, Václav Voráček^2*^, Radko Souček^5^, Kosuke Fujishima^3^, and Klára Hlouchová^1,5*^

^1^ Department of Cell Biology, Faculty of Science, Charles University, Biocev, Prague, Czech Republic.

^2^ Center for Machine Perception, Department of Cybernetics, Faculty of Electrical Engineering,Czech Technical University in Prague, Technicka 2, 166 27, Prague, Czech Republic

^3^ Earth-Life Science Institute, Tokyo Institute of Technology, Tokyo, 1528550, Japan

^4^ Department of Biochemistry, Faculty of Science, Charles University, Hlavova 8, 128 00, Prague 2, Czech Republic.

^5^ Institute of Organic Chemistry and Biochemistry IOCB Research Centre & Gilead Sciences, Academy of Sciences of the Czech Republic, Flemingovo nám. 2, 166 10, Prague, Czech Republic

*correspondence: [klara.hlouchova@natur.cuni.cz](mailto:klara.hlouchova@natur.cuni.cz), [voracva1@fel.cvut.cz](mailto:voracva1@fel.cvut.cz)

Supporting Information

**Sequences**

DNA library template is comprised of degenerate stretch of 99 nucleotides. Degenerate nucleotides are defined by IUPAC nucleotide code.

>library

CTGTAATACGACTCACTATAGGGACACCAATAGAGAAAGAGGAGAAATACTAGATGHYAGVYVTTGVMHYAGHABYAGVWHYABYGVTYHYAGHMHYAHYAHYAWYABYRHCKHYAWYAGVVGVHGVRGHVGDNGHAGDWGDMABTHCAGVBGVYAAAAGCCACCACCACCACCACCATCATCATCAGCATTAATAG

>reverse

CTATTAATGCTGATGATGATGGTGGTGG

>expressed protein

M(33-amino acid randomized region)KSHHHHHHHHQH

**Supporting Tables**

**Supporting Table S1.** Amino acid distributions used to benchmark CoLiDe

**Supporting Table S2.** Summary of CoLiDe benchmarking results. Precision is presented as the mean of 10 mean squared errors from solutions for each library length. Variation is described by the coefficient of variation for 10 solutions.

**Supporting Table S3.** CoLiDe Performance analysis – SwiftLib / CoLiDe template comparison

# Supporting Table S4. CoLiDe Performance analysis - Comparison of solutions for 4 inputs provided by CoLiDe (multiple codon set) and SwiftLib

# Supporting Table S5. CoLiDe Performance analysis - Comparison of solutions for 4 inputs provided by CoLiDe (restricted codon set) and SwiftLib

# Supporting Table S6. Experimental library - Summary of amino acid distributions

# Supporting Table S7. Experimental library - DNA and mRNA sequencing statistics

**Supporting Figures**

**
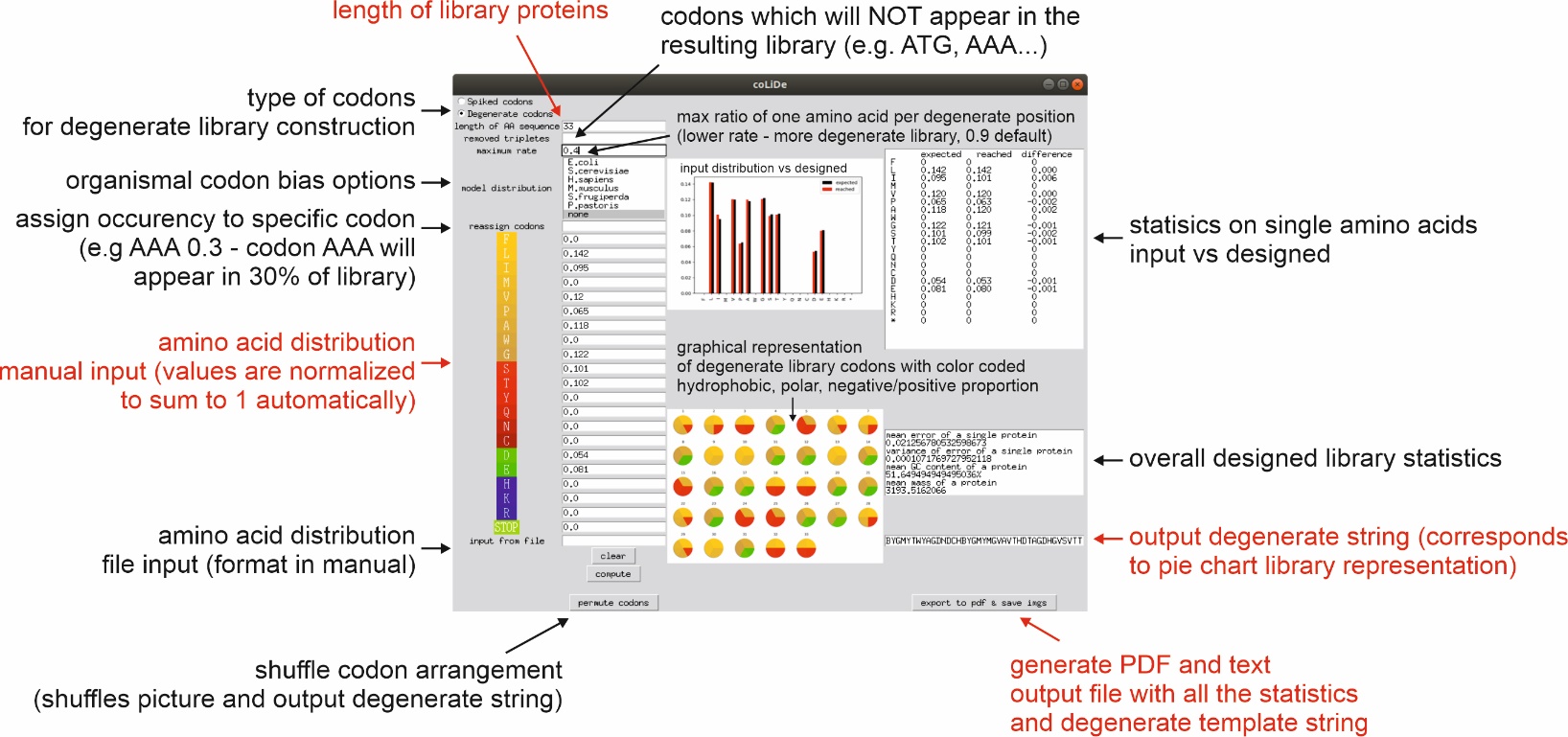
**

**Supporting Figure S1.** Main window of degenerate codon optimization tool CoLiDe. Input and output controls are in red

**
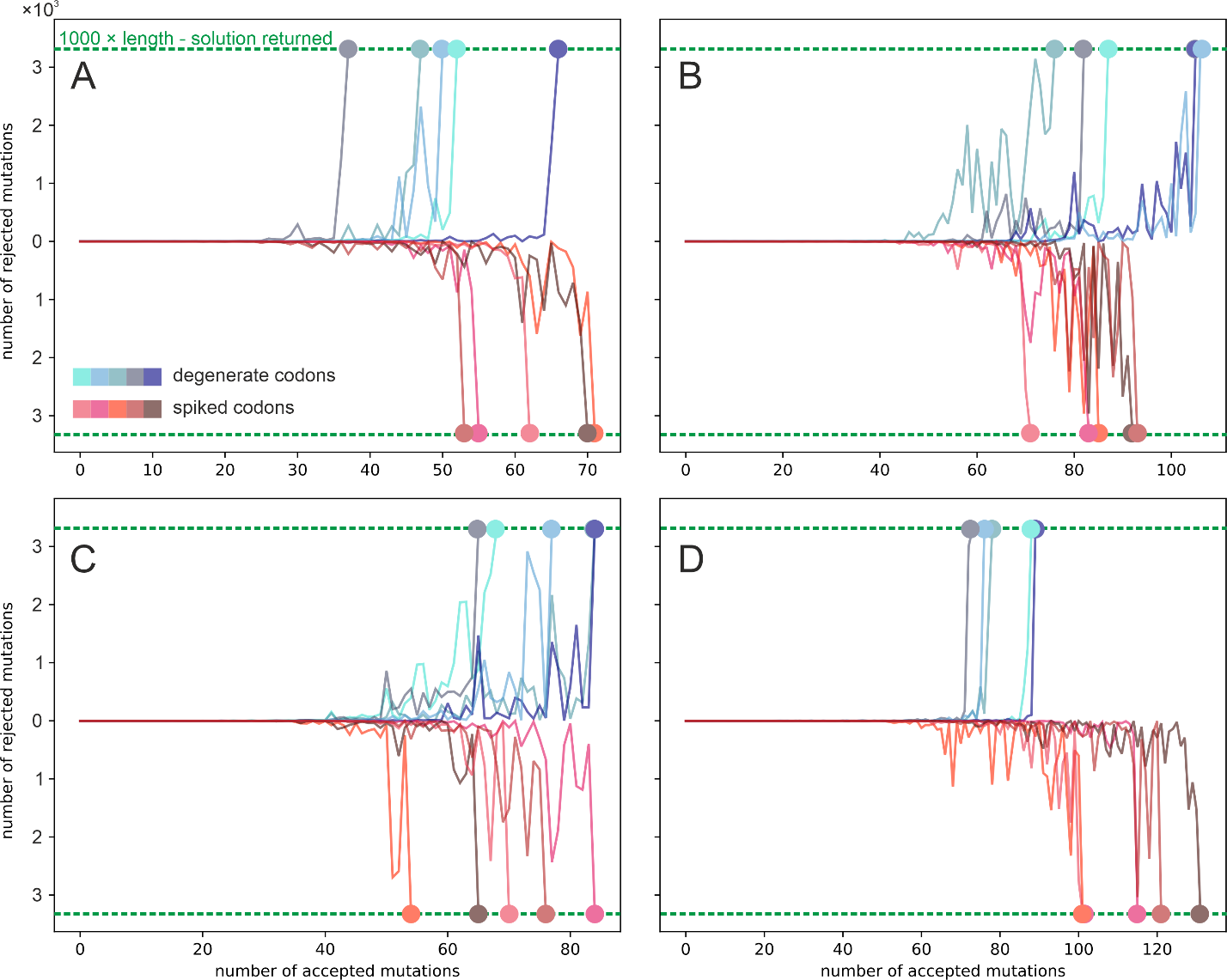
**

**Supporting Figure S2.** Acceptance/rejection statistics from CoLiDe optimization of early (A), RNA binding (B), natural (C) and rational (D) library lengths of 33 codons. All distributions were solved 5 times with either degenerate (blue) and spiked (red) codons. The analysis demonstrates an increasing rejection rate of mutations as solutions are being optimized towards the input distribution. The calculations converged after 33 000 (33 × 1 000) rejections after last accepted mutation was registered (green dashed line)


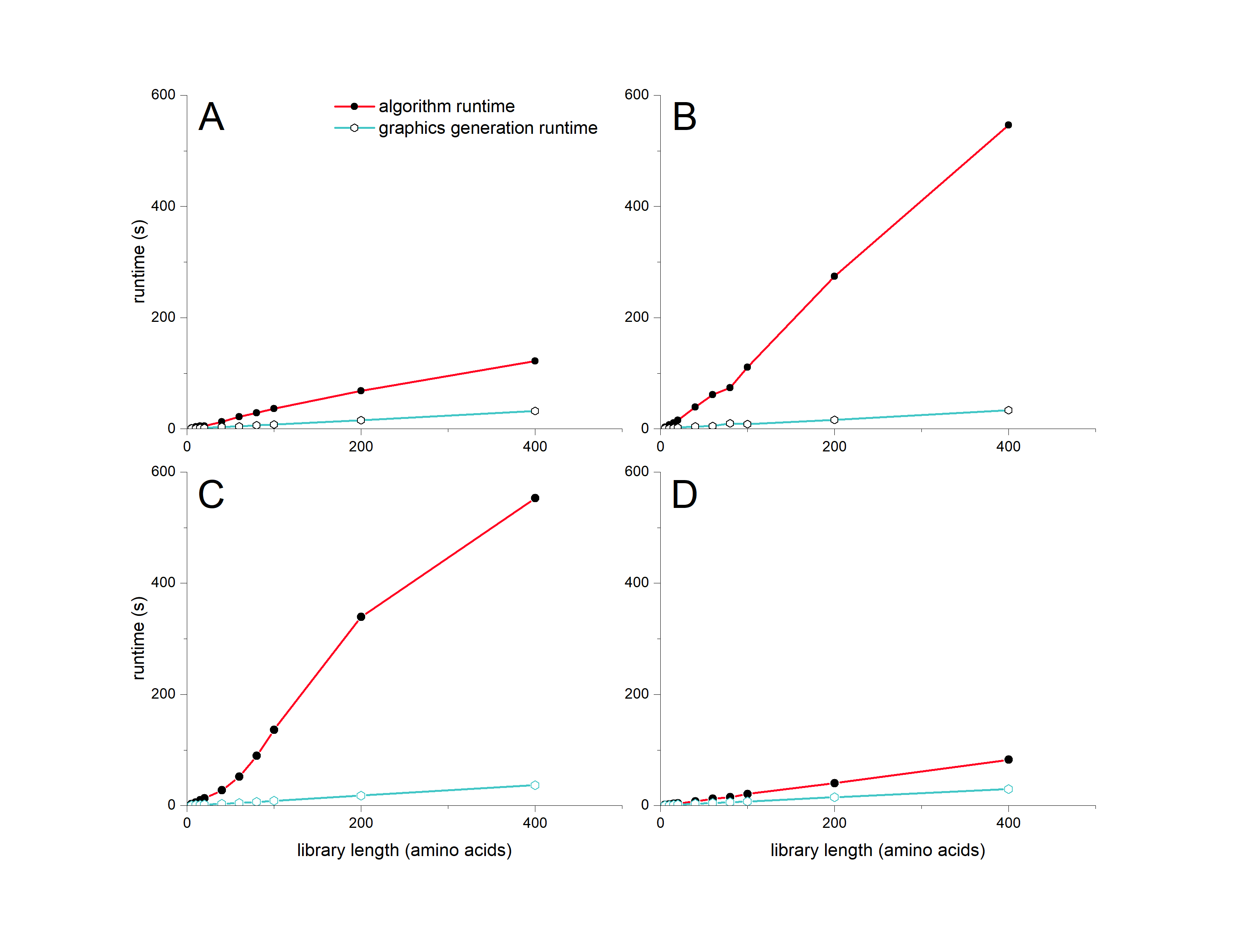
**Supporting Figure S3.** CoLiDe runtimes on (A) early, (B) RNA binding, (C) natural and (D) rational library designs. Runtimes were calculated by averaging of 10 measures on each library size ranging from 5 to 400 amino acids. Data were generated on Intel i5-8250U laptop


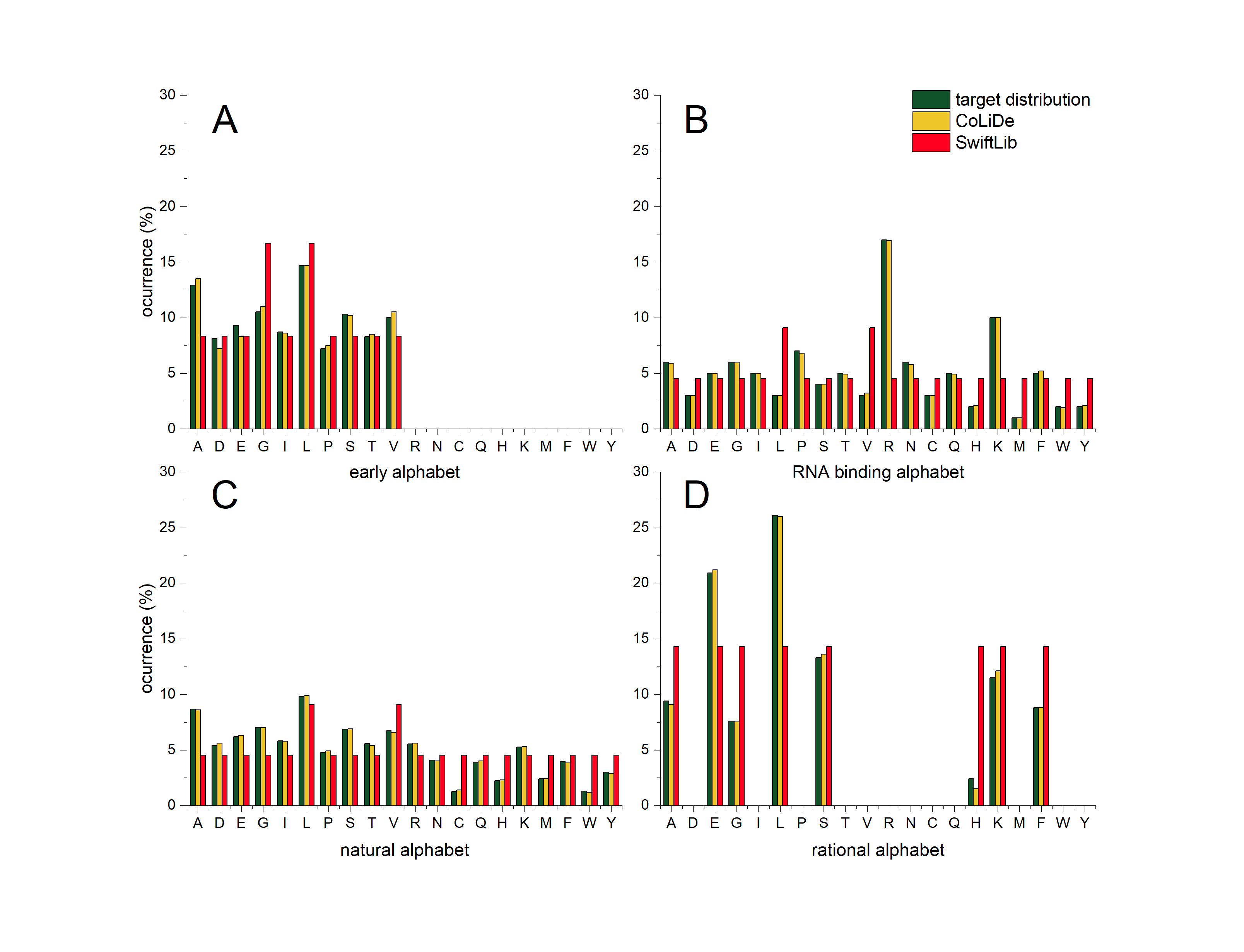


**Supporting Figure S4.** Amino acid distributions by CoLiDe (yellow) and SwiftLib (red) on four different amino acid alphabets (green). CoLiDe solutions for 33 amino acid libraries is compared for 2 (A,D) and 3-codon (B,C) distributions provided by SwiftLib

**
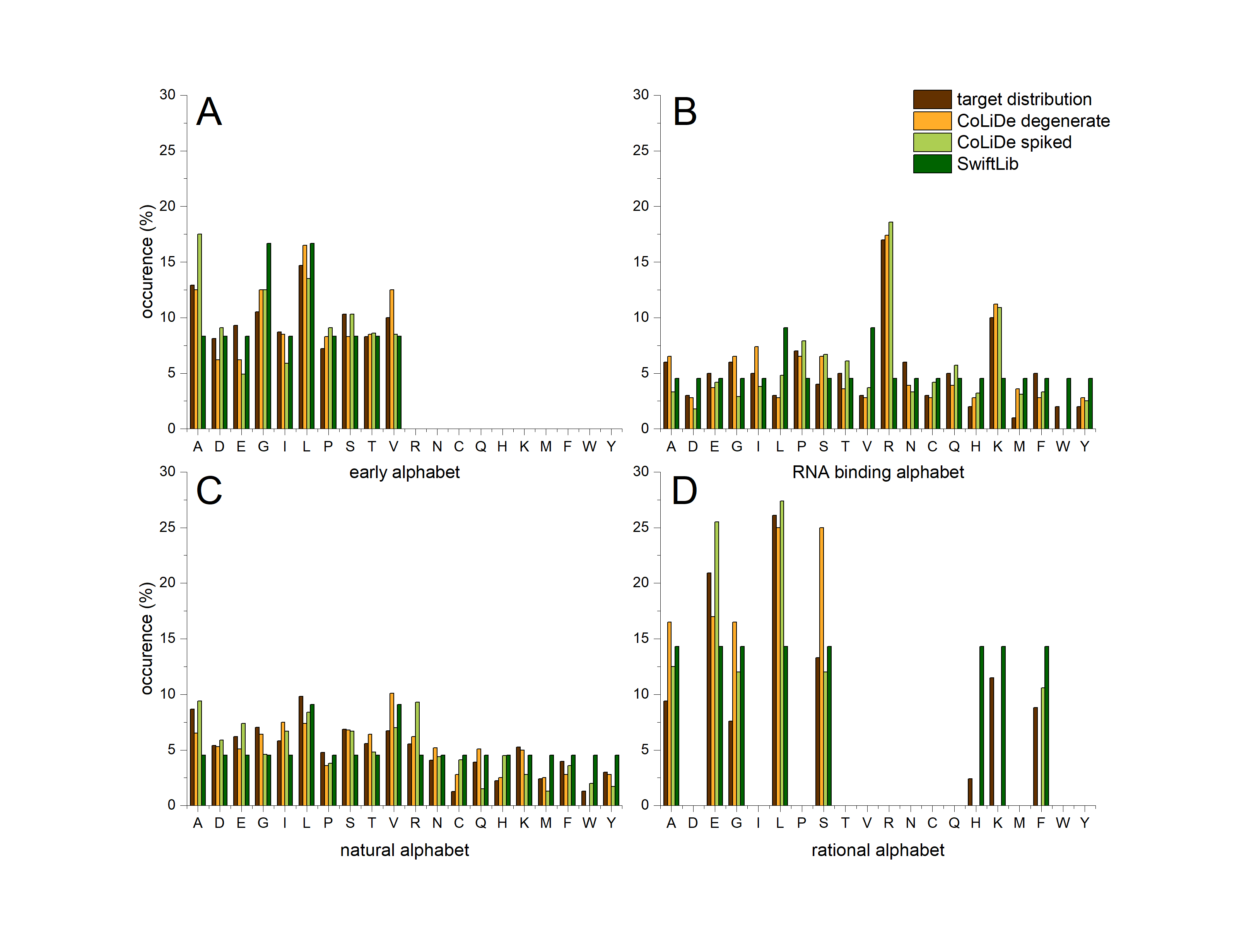
Supporting Figure S5.** Amino acid distributions by CoLiDe degenerate (orange), CoLiDe spiked routine (light green) and SwiftLib (green) on four different amino acid alphabets (green). CoLiDe was set to approximate input alphabets on 2 (A,D) and 3 (B,C) codons as equal to SwiftLib

**
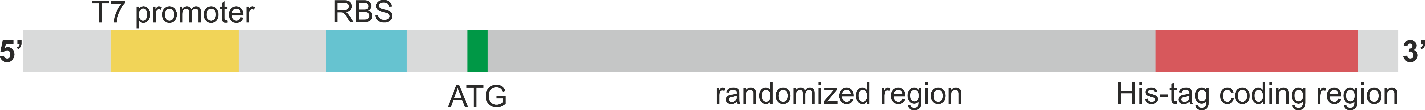
**

**Supporting Figure S6.** Design of the linear random library expression cassette. The cassette includes a randomized region of 33 amino acids along with sequences for *in vitro* transcription/translation and affinity purification of proteins using 8× His-tag.

**
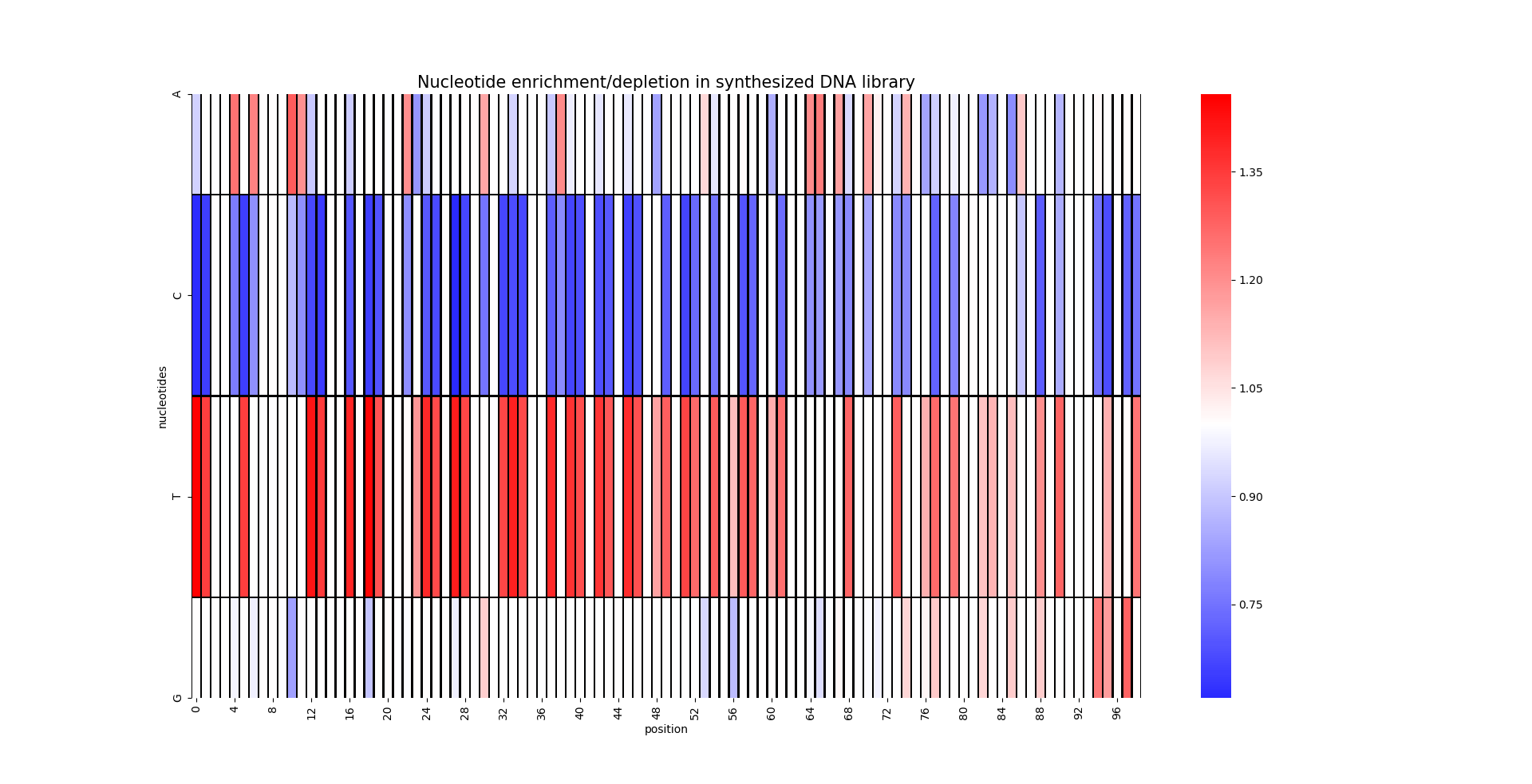
Supporting Figure S7.** Analysis of nucleotide enrichment/depletion in comparison to the designed template. Data were generated by analysis of 600,000 sequences obtained by HTS and averaging the frequencies of nucleotides in degenerate positions. Frequencies were compared with theoretical nucleotide occurrence probabilities. Deviations are visualized as heatmap of ratio p(sequencing)/p(theoretical), where p stands for the probability of nucleotide occurrence.
